# Key Components for Antibiotic Dose Optimization of Sepsis in Neonates and Infants

**DOI:** 10.3389/fped.2018.00325

**Published:** 2018-10-29

**Authors:** Tamara van Donge, Julia A. Bielicki, John van den Anker, Marc Pfister

**Affiliations:** ^1^Paediatric Pharmacology and Pharmacometrics, University Children's Hospital Basel, University of Basel, Basel, Switzerland; ^2^Paediatric Infectious Diseases Research Group, Institute for Infection and Immunity, St George's, University of London, London, United Kingdom; ^3^Intensive Care and Department of Paediatric Surgery, Erasmus MC-Sophia Children's Hospital, Rotterdam, Netherlands; ^4^Division of Clinical Pharmacology, Children's National Health System, Washington, DC, United States; ^5^Certara LP, Princeton, NJ, United States

**Keywords:** antibiotics, empirical phase, exposure, neonates, targeted phase, sepsis

## Abstract

Sepsis in neonates and infants remains a major cause of death despite a decline in child mortality and morbidity over the last decades. A key factor in further reducing poor clinical outcomes is the optimal use of antibiotics in sepsis management. Developmental changes such as maturation of organ function and capacity of drug metabolizing enzymes can affect the pharmacokinetic profile and therefore the antibiotic exposure and response in neonates and infants. Optimal antibiotic treatment of sepsis in neonates and young infants is dependent on several key components such as the determination of treatment phase, the administered dose and the resulted drug exposure and microbiological response. During the initial phase of suspected sepsis, the primary focus of empirical treatment is to assure efficacy. Once bacterial infection as the cause of sepsis is confirmed the focus shifts toward a targeted treatment, ensuring an optimal balance between efficacy and safety. Interpretation of antibiotic exposure and microbiological response in neonates and infants is multifaceted. The response or treatment effect can be determined by the microbiological parameters (MIC) together with the characteristics of the pathogen (time- or concentration dependent). The antibiotic response is influenced by the properties of the causative pathogen and the unique characteristics of the vulnerable patient population such as reduced humoral response or reduced skin barrier function. Therapeutic drug monitoring (TDM) of antibiotics may be used to increase effectiveness while maximizing safety and minimizing the toxicity, but requires expertise in different fields and requires collaborations between physicians, lab technicians, and quantitative clinical pharmacologists. Understanding these clinical, pharmacological, and microbiological components and their underlying relationship can provide a scientific basic for proper antibiotic use and reduction of antibiotic resistance in neonates and infants. This highlights the necessity of a close multidisciplinary collaboration between physicians, pharmacists, clinical pharmacologists and microbiologist to assure the optimal utilization of antibiotics in neonates and young infants.

## Introduction

Despite a decline in child mortality during the last decades, close to 6 million children died before the age of 5 years in 2015 with almost half of these patients dying during the neonatal period (1). Neonates are immunologically immature, have reduced skin barrier, reduced humoral response and a diminished microbial diversity in gut microbiota, all contributing to a higher risk of life-threatening bacterial infection, often presenting as sepsis (2–5). Sepsis is defined as a clinical condition resulting from a dysregulated immune response, triggered by an infection. The initiation of the pro-inflammatory cascade may cause widespread tissue injury (6–9). In 2015, infectious diseases were responsible for 9.5% of neonatal deaths worldwide, mainly focusing on lower and middle income countries where healthcare and appropriate antibiotics may be difficult to access (1). It should be noted that sepsis continues to impact not only neonates, but also affects a considerable proportion of young and older infants receiving intensive care. A recent study showed that global prevalence of severe sepsis in pediatric intensive care units is 8.2% (10).

The diagnosis of sepsis in neonates and infants is complex, and a complete discussion on clinical decision-making about initiations of antibiotics is beyond the scope of this review (11). Early antibiotic therapy for potential bacterial infection in sepsis is critical with antibiotics generally being started empirically, meaning before microbiological results are available. Antibiotic treatment is often started before sepsis is confirmed by microbiological diagnostics because of the lack of sensitive blood cultures together with the insufficient predictive performance of these analytics and as well as the possibility of sampling from the infection site. In settings with restricted availability of standard diagnostic tools or a high level of prior antibiotic exposure, for example because of availability of antibiotics over the counter, a definitive diagnosis may not be reached (12).

Neonatal sepsis can be divided into early and late onset neonatal sepsis (EONS and LONS), which reflects the timing of onset of symptoms, type and virulence of organism and associated pathogenesis (2). First, EONS is defined by a life-threatening infection during the 1 days of life. In developed countries Group B *Streptococcus* and *Escherichia coli* account for most episodes of EONS, whereas *Klebsiella* is the most common organism in low and middle income countries (13, 14). Risk factors for EONS are prematurity, premature and prolonged rupture of membranes, intrapartum maternal fever (>38°C) and maternal Group B *Streptococcus* colonization (3, 15, 16). As expected, neonates with a very low birth weight (VLBW, <1,500 g) are more susceptible to an infection (16, 17). Second, LONS is characterized by the onset of symptoms more than 72 h after birth. Among VLBW neonates, Gram-positive organisms are most commonly associated with LONS, although it has been shown that the mortality rate is 2–3 times higher in neonates with Gram-negative infections. Prolonged indwelling catheter use and other invasive procedures are potential risk factors (16). Third, invasive infections during infancy are mostly caused by *Streptococcus pneumoniae*. Because of vaccinations, infections caused by *Haemophilus influenzae* type b are less common in developed countries compared to resource limited settings (7). Currently, *Salmonella* spp. is one of the most common organisms causing sepsis in low and middle income countries (18).

**Figure 1:**
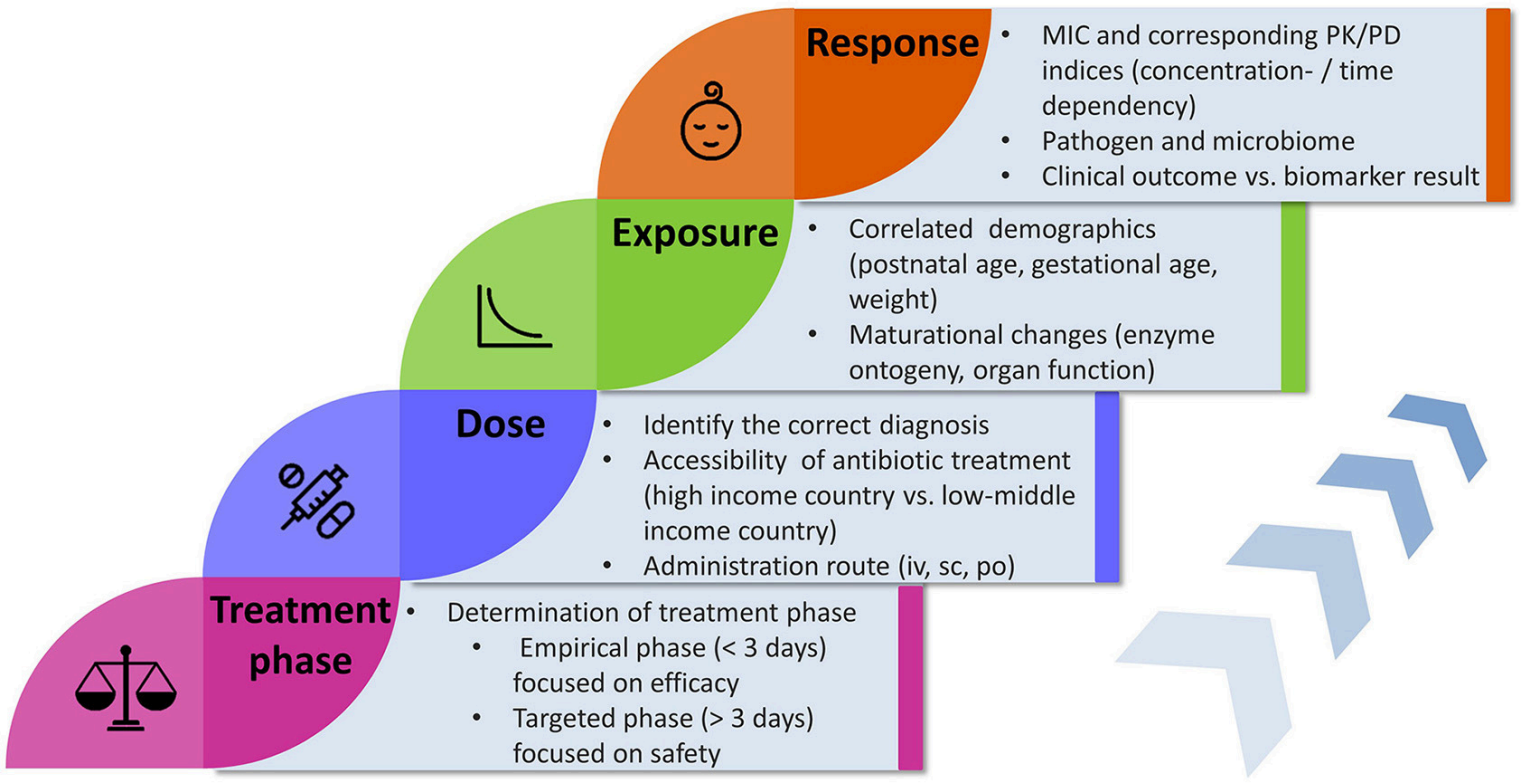
Flowchart illustrating key components and factors influencing the concepts concerning antibiotic treatment in neonates and infants. MIC, minimal inhibitory concentration; PK, pharmacokinetics; PD, pharmacodynamics; iv, intravenous; sc, subcutaneous; po, oral.

In the first 2 years of life, maturational processes affect drug clearance and make antibiotic dosing more challenging, compared to older infants where dosing is mainly adjusted by body weight and renal function. Most of the current dosing guidelines for antibiotic treatment are simply extrapolated from adult studies and it has been reported that dosing recommendations across intensive care units and international guidelines are highly variable and inconsistent (19). We review and discuss key components and their underlying relationships relevant to antibiotic dose optimization in neonates and infants with suspected or confirmed sepsis (Figure 1).

## Optimization of antibiotic therapy in sepsis: empirical vs. targeted treatment

**Figure 2:**
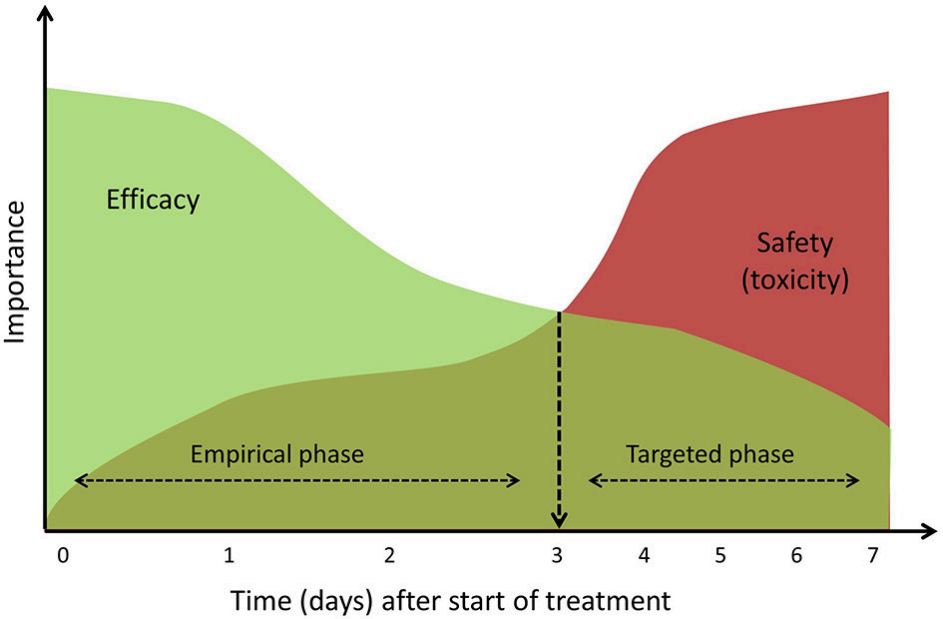
Conceptual visualization of impact of efficacy and toxicity during antibiotic treatment. During the empirical phase, focus lies on an efficacious treatment and when sepsis is confirmed (~3 days) treatment can be individualized and focus should shift to the safety of treatment.

In a clinical setting, there is generally no time to wait for the result from microbiologic samples when there is suspected sepsis. Antibiotic treatment can therefore be viewed as having two phases, namely an initial, empirical treatment phase followed by a targeted treatment phase once a causative pathogen is confirmed (Figure 2). Both phases are time-related, and antibiotic dose optimization may focus on either efficacy or safety, respectively.

### Empirical treatment phase

In the 1 hours to days of treatment, the primary focus is to deliver effective treatment. During this earliest stage mortality is directly related to the effects of the life-threatening infection and managing toxicity is less central. As the causative organism generally remains unknown, selection of the antibiotic regimen needs to take into account the overall epidemiology of sepsis in the age group of the patient (19).

A key parameter describing susceptibility to antibiotics and used in dose-finding is the minimal inhibitory concentration, or MIC, which reflects the lowest antibiotic concentration needed to inhibit visible growth of the pathogen (20). MIC breakpoints for pathogens are established based on various *in vitro* tests and are applied to an entire population. Initial antibiotic doses should be targeting the “worst-case” minimal inhibitory concentrations, captured by the phrase “go hard and go home” (21). During the empirical treatment phase, the benefits (e.g., high probability that causative pathogens are killed) outweigh the risks (e.g., development of renal toxicity) and therefore a certain trade-off in dosing regimen to achieve relatively high exposures in relation to non-pathogen specific MIC may be acceptable.

### Targeted treatment phase

After an initial empirical treatment there are two possible outcomes. Treatment may be discontinued because the clinical picture of sepsis cannot be microbiologically confirmed and an alternative diagnosis emerges. On the other hand, the microbiological cause confirming the diagnosis of sepsis may be identified. In the latter case treatment will be continued and toxicity issues become more important. During this targeted treatment phase, antibiotic dose optimization will be individualized to achieve an optimal efficacy-safety balance (Figure 2). When patients experience or are at high risk of toxicity (for example because of renal failure), three options are available: if susceptibility testing suggests a less toxic alternative, antibiotic treatment may be switched; depending on the exact infection and treatment response, only a short course is necessary and treatment may be stopped; or the antibiotic is question is considered the optimal therapeutic choice, in which case dose adjustments will be needed, possibly combined with therapeutic drug monitoring (TDM).

### Antibiotic drug monitoring

The relationship between antibiotic dose and exposure is subject to high levels of inter- and intra-individual variability and to achieve effective antibiotic exposure, antibiotic drug monitoring is becoming crucial. This variability is known to be increased in patients with life-threatening infection, when rapid pathophysiological fluctuations even over the course of a few hours can impact the pharmacokinetics, and therefore the relationship between dose and antibiotic exposure. Reliable measurements are a prerequisite for effective TDM, accordingly turn-around times >24 h should be disregarded for critically ill patients (22). TDM is used to personalize the dosing strategies to ensure antimicrobial exposures which have therapeutic success and low probabilities of toxicity and generation of antimicrobial resistance (23). The percentage of patients with sub-therapeutic concentrations decreased from 58 to 40% after applying TDM for vancomycin in preterm and term neonates (24). Adequate antibiotic drug monitoring requires expertise in different fields and calls for the collaboration of physicians together with the lab technicians and clinical pharmacologists.

While the above is likely to be applicable to any antibiotic treatment, different antibiotics have different characteristics which are reflected in their pharmacological behavior. Most β-lactams have a wide therapeutic window, meaning that even high exposure is unlikely to be associated with toxicity. In contrast, aminoglycosides and glycopeptides have a narrow therapeutic window and require more attention to avoid toxicity.

## Understanding dose, drug administration, exposure, and response

**Figure 3:**
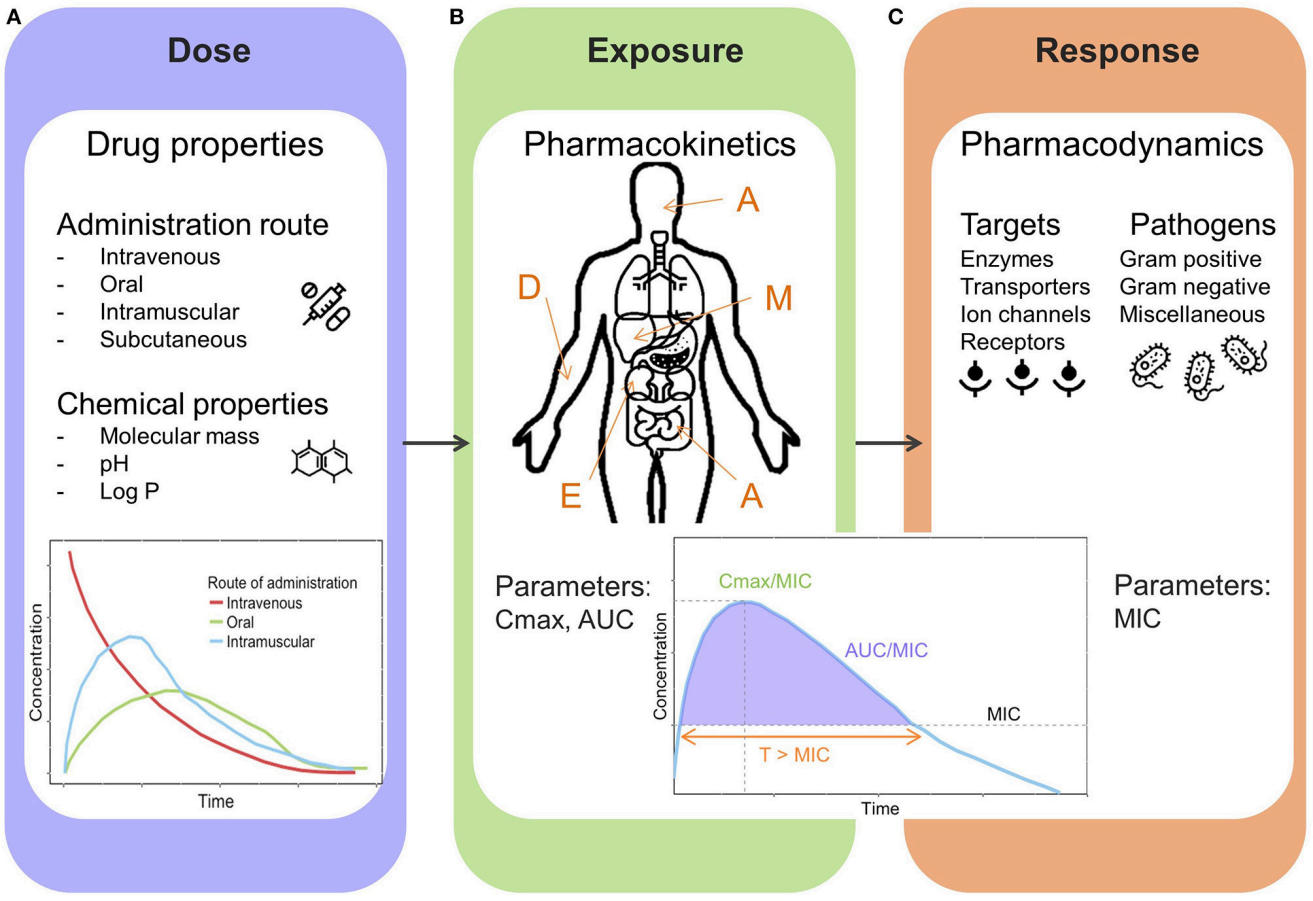
General overview illustrating pharmacological key components. **(A)** Dose: drug properties and administration routes. **(B)** Exposure: Pharmacokinetic processes and parameters, A, Absorption (e.g., intestines); D, Distribution (e.g., blood circulation); M, Metabolism (e.g., liver); E, Excretion (e.g., kidneys); Cmax, peak concentration; AUC, area under the concentration-time curve. **(C)** Response: Pharmacodynamics, targets and pathogens, MIC, minimal inhibitory concentration; T > MIC, time above MIC.

Clinical pharmacology aims to predict both efficacy and safety based on drug properties, population or individual pharmacokinetic behavior (PK) and pharmacodynamic, microbiological characteristics (PD). In order to understand optimal and individualized dosing of antibiotic treatment, one should be aware of the drug related processes in the human body and their influences on each other (Figure 3).

### Dose and drug administration

A drug can have several formulations and can be administered through various routes, intravenous and oral being the most frequently used (Figure 3A). However, for early treatment of neonatal sepsis, oral administration is not clinically relevant. The route and method of administration can influence both PK and PD processes, and therefore needs to be considered when determining optimized dosing recommendations. Aminoglycosides are mostly administered via intravenous bolus dosage to achieve effective peak concentrations, due to concentration dependent properties. In countries where healthcare may be difficult to access, intramuscular administration is often applied.

### Drug exposure

The relationship between dose and drug exposure is governed by pharmacokinetics, defined by the kinetic processes abbreviated as ADME, which defines the absorption, distribution, metabolism, and excretion of a drug (Figure 3B). Due to dynamic maturation processes neonates and infants have marked differences compared to adults in terms of physiology affecting the different pharmacokinetic stages (25, 26). The total body water in infancy is decreasing over time (80–90% compared to 55–60% in adults), which influences the distribution of water soluble drugs such as gentamicin. Drug eliminating organs such as liver and kidney are immature at birth. During the first 2 weeks of life glomerular filtration rate increases rapidly reaching adult values within 1–2 years (27, 28). The metabolic capacity is determined by the ontogeny of metabolizing enzymes (a majority of them located in liver). Generally, the rate of hepatic metabolism is low at birth and increases over time, depending on the type of enzyme. These processes have an impact on exposure of antibiotics, and therefore dosing needs to be adjusted based on demographic characteristics of an individual neonate or infant.

### Microbiological response

Pharmacodynamic (PD) and microbiological aspects focus on the effects of a given drug on the pathogen and body (Figure 3C). In order to elicit an effect, antibiotics need to reach certain exposure levels to kill causative pathogens of a sepsis. The exposure induced by the antibiotic dose will cause a response, but the main target being the pathogen. Currently, the MIC-based approach is most frequently applied to link drug exposure to microbiological response (Figure 3C).

Understanding the PK of antibiotics is necessary but not sufficient for optimizing and individualizing dosing strategies. It is essential to also understand characteristics and dynamics of the target (pathogen) as well (29). The growth of the pathogen needs to be inhibited or, even better, stopped entirely by the antibiotic agent depending on the MIC (Table 1). However the MIC may not be a fixed value, but rather changes over time, for example in the context of antibacterial resistance, and is also subject to measurement errors to the test system (variations in pH, incubation time, etc.) (38).

**Table 1:** Pharmacokinetic and pharmacodynamic indices for antimicrobial agents together with their target value and bactericidal characteristics.

**PK/PD indices and their target values**				
**Antimicrobial agents**	**PK/PD index**	**Target value for clinical antibacterial efficacy**	**PK/PD properties**	**References**
Aminoglycosides Gentamicin Amikacin Tobramycin	Cmax/MIC	≥8 8–10 8–12	Concentration dependent killing (maximize drug concentration)	(22) (30) (31)
β-lactams Penicillins Carbapenems Cephalosporins	fT > MIC	T>MIC > 40% T>MIC > 50–60% T>MIC > 40–50% T>MIC > 60–70%	Time dependent killing (maximize exposure time)	(32) (33) (33) (33)
Glycopeptides Vancomycin Quinolones Levofloxacin Ciprofloxacin Fluoroquinolones	AUC24/MIC AUC/MIC AUC/MIC AUC24/MIC	400 100 125 100–125 (Gram-negatives) 25–35 (Gram-positives)	Time and concentration dependent killing (maximize daily amount of dose)	(34) (35) (36) (37)

*Cmax: maximum antibacterial concentration, MIC: minimal inhibitory concentration, AUC: area under the concentration-time-curve, AUC24: area under the concentration time curve over 24 hours, fT > MIC: percentage of time for which the free fraction of drug remains above MIC*.

An increase in MICs, which is the result of decreasing susceptibility of a pathogen in a population, must in many cases be accompanied by dose adjustments to ensure effective exposure and maximize the effect. Recent changes to the interpretation of the so-called intermediate breakpoint as representing susceptibility for which successful treatment outcomes are likely with adjustments of the dosing regimen reflect this (10).

## Understanding the link between antibiotic exposure and microbiological response

With a limited pipeline of new antibiotics, relying on proper use and understanding the link between antibiotic exposure (PK) and microbiological response (pharmacodynamics, PD) is a key issue concerning dosing optimization of the presently available antibiotics (29).

In order to describe relationships between drug exposure and microbiological effects, exposure-response parameters are used. A PK/PD index is defined as the quantitative relationship between an exposure-related parameter (e.g. plasma concentration) and a microbiological parameter (e.g. MIC) (39). Antibiotic classes can be characterized by different properties in terms of PK/PD indices. The optimal target index is frequently identified based on animal dose fractionation studies (37). Already in the early 1950s Eagle *et al*. noticed the time dependent properties of penicillin, and realized that penicillins are best administered as continuous infusions, whereas a concentration dependent agent is better given as an intravenous bolus to achieve high maximum concentrations (40, 41).

### Concentration dependent microbiological response

The bacterial killing rate of concentration dependent antibiotics increases at high levels of the antibiotic; this applies to aminoglycosides and fluoroquinolones. For aminoglycosides, the antibacterial effect is related to the peak concentration (*C*_*max*_*/MIC*). Depending on the antibiotic class, different ratios apply (Table 1). The magnitude of the peak concentration is often associated with the bacterial killing efficiency (go hard and go home paradigm) (21, 40, 42). In addition, concentration dependent antibiotics frequently exhibit a post antibiotic effect (PAE). The PAE is defined as the suppression of bacterial growth after the exposure of bacteria to an antibiotic (even in absence of host defense mechanism) (43).

Shifts in MIC can lead to a situation where dosing recommendations need to be revised to achieve optimal treatment. For gentamicin, for example, an increase in MIC from 0.5 to 1.0 mg/L means that, in order to achieve similar efficacy (similar ratio of *Cmax/MIC*), the dose should be increased from 5 to 7.5 mg/kg in neonates (19).

### Time dependent microbiological response

The effect of time dependent antibiotics relies on the length of time that the antibiotic is in contact with causative pathogen. For β-lactams the antibacterial effect is considered to be time dependent and therefore the PK/PD index *Time/MIC* is used (Figures 3B,C). This index is generally transformed to *fT*>*MIC;* this reflects the percentage of time for which the free fraction of drug concentration remains above the MIC (Table 1). For β-lactams (penicillins, cephalosporins, carbapenems) it has been proposed that dosing schedules should maintain plasma concentrations above MIC for at least 50% of the dosing interval, but the efficacy of β-lactams is enhanced with longer exposure times. The post antibiotic effect is limited for β-lactams with an exception for carbapenems (40). Continuous infusions can potentially improve target attainment for *fT*>*MIC*, they may, however, be impractical in many settings (44). Decreased mortality has been associated with continuous infusion of β-lactam antibiotics in critically ill patients with severe sepsis (45).

### Other relevant indices for microbiological response

Several studies have shown the importance of a third index, namely *AUC/MIC* (21, 38). AUC reflects the area under the concentration-time curve and represents the antibiotic exposure over time. This parameter is often used for concentration independent antibiotics with extended post antibiotic effects, such as vancomycin. Bacterial regrowth is inhibited, even when the concentration falls below MIC, but the effect is not dependent on the peak concentration (37). Few antibiotics, such as aminoglycosides and fluoroquinolones have been linked to multiple classes and multiple corresponding indices, leading to differences in dosing recommendations and guidelines.

## Challenges of antibiotic dose optimization in neonates and infants

Currently, TDM of antibiotics is not widely used for antibiotic dose optimization in neonates and infants suffering from life-threatening infections. This is mainly related to practical barriers of implementing TDM for improving treatment effectiveness, such as the lack of rapid and reliable methods of analysis of the antibiotic or the possibility that the pharmacologic effect is not readily measurable (due to interactions with other drugs) (46) Beta-lactam antibiotics in particular would benefit from dose adaptations based on measured levels, as these are often the backbone of empiric treatment (47). Technical bottlenecks include long turn-around times for samples, lack of commercial assays and challenging pre-analytics, and in the pediatric population the need for relatively large samples volumes (Table 2). The required sampling volume, relative to the circulating blood volume is a crucial barrier, especially in preterm infants whose blood volume is limited. Furthermore, concentration measurements are often collected from plasma since these are relatively easy to obtain, although these levels appear to be a poor descriptor of the activities of the drug at site of action in individual patients.

**Table 2:** Challenges to overcome the burden of sepsis and the opportunities to improve diagnostic tools, measurement techniques and implementation of modeling and simulation techniques.

**Challenges; what is missing?**	**Opportunities**
• Uniform sepsis definitions for all age groups across the pediatric age range	• Identify of biomarkers (e.g., presepsin or cystatin C) with accurate thresholds
• Diagnostic tools to identify pathogens and infection	• Use microdialysis to measure drug concentrations at target site
• Adequate descriptors of drug concentration at target site	• Implement therapeutic antibiotic monitoring, especially in patients with life-threatening infections
• Understanding PK/PD relationships and parameters which can characterize the dynamic process of antibacterial activity	• Apply kill-curves approach to describe changing antibacterial activity
• Reliable measurements for GFR in the pediatric population (augmented renal clearance)	• Multidisciplinary collaboration and communication between research groups and physicians
• Straightforward applications of model-based approaches	• Implement modeling and simulation strategies in clinical settings (e.g., for individual dose optimization)
• Implementation of adjusted dosing guidelines in clinical practice	• Develop understandable time-saving software tools for individualized dosing

Moreover, although the MIC-based approach is well-established as a measure of the potency of an antibiotic drug, it is determined in an *in vitro* setting, where the conditions are dissimilar from those at the site of infection in the *in vivo* situation. Better understanding of population-specific MICs is demanded to guide empiric antibiotic treatment (40, 48). Additionally, antibacterial activity is a dynamic process and since MIC is a one-point threshold value, the MIC can only provide an approximation on the antibacterial effect (38).

The key issue in optimizing antibiotic exposure in critically ill patients is to respond to expectedly variable PK in patients with life-threatening illness and at risk of infection caused by bacteria with potentially problematic antibiotic resistance. Critical illness leads to time-variation in multiple factors, potentially requiring frequent dose adjustments in the most vulnerable patients rather than simple *a priori* dose stratification. More knowledge is required concerning tissue penetration of antibiotics in critically ill neonates and infants (49). Furthermore, drug dosing is currently being adjusted for patients with impaired kidney function (risk for toxicity), whereas for patients with augmented renal clearance (elevated drug clearance) no dose adjustments are being recommended (50). Although the underlying physiological mechanisms of augmented renal clearance are not yet fully understood, augmented renal clearance has not only been observed in critically ill adults, but also in pediatric patients (50, 51). Consequently, there is a real need for reliable assessment and monitoring of kidney function in neonates and infants. Serum creatinine values are still widely used, although the accuracy and usefulness of this biomarker can be questioned in neonates as various parts of kidneys are maturing at different rates (52).

The application of pharmacometric modeling and simulation will be needed to truly support antibiotic dosing optimization based on the knowledge of the dose-concentration-effect relationship (53). The modeling and simulation strategy is still underutilized, although it has been shown that mechanistic modeling such as physiological-based pharmacokinetic (PBPK) models have good predictive value and enable extrapolation by using information about the drug and the physiology (54, 55). Despite modeling and simulation being frequently reported in the literature, the results and adjusted dosing recommendations are not yet implemented in daily clinical practice (56). Dose adjustment and individualization of antibiotics is crucial. For instance, administration of an inefficacious (too low) dose of antibiotics in patients with increased drug clearance can have a negative impact on patient outcome and antibiotic resistance.

## Opportunities: how to close knowledge gaps

The search for a quantitative, scientific rationale to further enhance dosing regimens and drug combinations can benefit tremendously from modeling and simulation strategies when there is on-going communication and exchange between research groups and clinicians (Table 2) (57). In order to apply these quantitative methods directly in clinical practice, it is essential to communicate the strengths and applicability of the model to the users (mostly physicians). User-friendly decision support tools, which provide quantitative, scientific output without requiring additional time-consuming activities during routine clinical practice, would be valuable (54, 56). An example of these software tools is the model-supported TDM tool for precision dosing TDMx (http://www.tdmx.eu/). Since there are several population PK models published for antimicrobial agents, researchers should assess new data or use existing data to extend and improve existing population PK models (56, 58). Pharmacometric PK/PD models can help identify the optimal (effective and safe) therapeutic window necessary to successfully treat an infection (59).

In contrast to the MIC, which reflects the susceptibility of a pathogen at only one time point, bacterial kill-curves can offer more detailed information about the killing activity as a function over time and might even be used to identify the presence of resistant subpopulations (60, 61). Bacterial kill-curves are very labor intensive and until the method is automated and widely implementable, this approach might not be practical (61).

Furthermore, in recent decades novel non-invasive techniques have provided information about the process of target site distribution. Microdialysis provides direct measurement of concentrations of unbound antibiotics at the site of action when the site of infection is not the bloodstream (40, 62). Measurement of the free (unbound) drug concentration in the interstitial fluid is better correlated with the antimicrobial efficacy, compared to concentration measurements in plasma. Microdialysis offers a useful sampling tool which can quantify the unbound antibiotic at infection sites (29). Other non-invasive techniques such as dried blood spot analysis or TDM from sweat are considered as innovative and promising methods to tackle the known barriers (30, 31, 34).

## Conclusions

There are still numerous challenges to overcome the burden of sepsis in neonates and infants, of which the lack of implementation of optimized, individualized dosing recommendations can be considered as remarkably important. Key components for optimal antibiotic treatment of sepsis in neonates and infants are indicated as treatment phase, dose, drug exposure and microbiological response. During the first days of treatment the focus lies on establishing an effective dose, thereafter the balance is shifting toward ensuring a safe and effective treatment. In neonates and young infants, drug exposure is affected by developmental changes such as maturation of organ function and metabolizing enzymes, which requires dosing adjustments. The response or treatment effect can be determined by the microbiological parameters (MIC) together with the pathogen characteristics (time- or concentration dependent). Understanding these clinical, pharmacological and microbiological components and their underlying relationship might provide a basis for proper antibiotic use and reduction of antibiotic resistance. This also illustrates the necessity of a close multidisciplinary collaboration between physicians, pharmacists, pharmacometricians, clinical pharmacologists and microbiologists to assure optimal utilization of antibiotics in neonates and infants.

## Author contributions

TvD, JB, and MP drafted the concept of the review. TvD wrote the first draft of the manuscript and generated tables and figures. JB contributed to the sections considering infectious diseases. JvdA and MP contributed to the clinical pharmacology section. All authors critically revised the manuscript, and approved the final version before submission.

### Conflict of interest statement

MP has a part-time employment with the consulting company Certara, USA. The remaining authors declare that the research was conducted in the absence of any commercial or financial relationships that could be construed as a potential conflict of interest.
